# Single-sided deafness and directional hearing: contribution of spectral cues and high-frequency hearing loss in the hearing ear

**DOI:** 10.3389/fnins.2014.00188

**Published:** 2014-07-04

**Authors:** Martijn J. H. Agterberg, Myrthe K. S. Hol, Marc M. Van Wanrooij, A. John Van Opstal, Ad F. M. Snik

**Affiliations:** ^1^Department of Biophysics, Donders Institute for Brain, Cognition and Behaviour, Radboud University NijmegenNijmegen, Netherlands; ^2^Department of Otorhinolaryngology, Donders Institute for Brain, Cognition and Behaviour, Radboud University Medical CenterNijmegen, Netherlands

**Keywords:** azimuth, head-shadow effect, mold, single-sided deaf(ness), spectral pinna-cues

## Abstract

Direction-specific interactions of sound waves with the head, torso, and pinna provide unique spectral-shape cues that are used for the localization of sounds in the vertical plane, whereas horizontal sound localization is based primarily on the processing of binaural acoustic differences in arrival time (interaural time differences, or ITDs) and sound level (interaural level differences, or ILDs). Because the binaural sound-localization cues are absent in listeners with total single-sided deafness (SSD), their ability to localize sound is heavily impaired. However, some studies have reported that SSD listeners are able, to some extent, to localize sound sources in azimuth, although the underlying mechanisms used for localization are unclear. To investigate whether SSD listeners rely on monaural pinna-induced spectral-shape cues of their hearing ear for directional hearing, we investigated localization performance for low-pass filtered (LP, <1.5 kHz), high-pass filtered (HP, >3kHz), and broadband (BB, 0.5–20 kHz) noises in the two-dimensional frontal hemifield. We tested whether localization performance of SSD listeners further deteriorated when the pinna cavities of their hearing ear were filled with a mold that disrupted their spectral-shape cues. To remove the potential use of perceived sound level as an invalid azimuth cue, we randomly varied stimulus presentation levels over a broad range (45–65 dB SPL). Several listeners with SSD could localize HP and BB sound sources in the horizontal plane, but inter-subject variability was considerable. Localization performance of these listeners strongly reduced after diminishing of their spectral pinna-cues. We further show that inter-subject variability of SSD can be explained to a large extent by the severity of high-frequency hearing loss in their hearing ear.

## Introduction

Listeners with total single-sided deafness (SSD) lack the ability to localize sounds on the basis of interaural differences in time (ITD) and sound level (ILD). As a result, SSD listeners encounter significant problems with the processing of auditory information in daily life (Van Wieringen et al., 2011; Lieu, 2013), and demonstrate impaired sound-localization abilities (Humes et al., 1980; Colburn, 1982; Slattery and Middlebrooks, 1994; Bosman et al., 2003; Van Wanrooij and Van Opstal, 2004; Wazen et al., 2005). Similar effects have been reported for unilateral plugged control listeners (McPartland et al., 1997; Van Wanrooij and Van Opstal, 2007; Kumpik et al., 2010; Irving and Moore, 2011; Agterberg et al., 2012), and unilateral plugged experimental animals (Keating et al., 2013; Kral et al., 2013). Several studies, in which sound levels were fixed or varied over a small range, have demonstrated sound-localization abilities of SSD listeners (Batteau, 1967; Colburn, 1982; Häusler et al., 1983; Slattery and Middlebrooks, 1994; Wightman and Kistler, 1997). When stimuli are presented at a single sound level, SSD listeners could rely on the perceived sound level at the hearing ear because of the azimuth-dependent attenuation produced by the head-shadow effect (HSE). Van Wanrooij and Van Opstal (2004) demonstrated that the HSE indeed contributes to sound localization abilities of SSD listeners. Furthermore, the possibility that these listeners have learned to use monaural pinna-induced spectral-shape cues of their hearing ear for localization in azimuth, has been postulated (Batteau, 1967; Colburn, 1982; Häusler et al., 1983; Slattery and Middlebrooks, 1994; Wightman and Kistler, 1997; Van Wanrooij and Van Opstal, 2004; Shub et al., 2008; Kumpik et al., 2010; Rothpletz et al., 2012). The studies mentioned above did not take into account the hearing loss of the better ear, and included only subjects with a normal hearing ear (i.e., hearing thresholds ≤25 dB HL at frequencies between 0.25 and 8 kHz). Especially when stimuli contain high-frequencies information, monaural pinna-induced spectral-shape cues can be beneficial for localization (Best et al., 2005). Recently it has been reported that older listeners (63–80 years) with hearing loss above 5 kHz demonstrated deteriorated sound localization in elevation as compared to normal hearing listeners (Otte et al., 2013). High-frequency hearing loss did not affect sound localization abilities in azimuth. These results show that with advancing age and subsequent increasing high-frequency hearing loss, listeners lose the access to spectral-shape information for the localization of broadband (BB) stimuli in elevation. The loss of this ability might be of importance for listeners with SSD.

Animal studies have indicated that early onset of unilateral deafness results in a unilateral aural preference, reflected by local field potentials recorded from the cortical surface (Kral et al., 2013). Others, demonstrated that the ability to use spectral localization cues diminished as soon as normal hearing was experienced (Keating et al., 2013). As it is unclear whether a critical period for this auditory plasticity might also be present in humans, and it is postulated that the etiology of subjects with SSD may be unrelated to their localization abilities (Colburn, 1982), we investigated whether the onset of unilateral deafness (congenital vs. acquired) affects sound-localization performance in azimuth and elevation when tested at a later age.

Listeners with SSD demonstrate a large variability in their localization performance and it is not clear whether this variation is related to hearing loss, pinna-induced spectral shape cues, or to the onset of unilateral deafness. In the present study we investigated to what extent high-frequency hearing loss in the hearing ear of SSD listeners affects their use of spectral-shape cues to localize sounds in azimuth. Furthermore, for SSD patients who are seeking hearing revalidation an improved number of treatment options have become available. It is important to identify the factors affecting sound localization abilities of SSD listeners. This information is helpful for clinicians in the search for the best possible treatment for listeners with monaural hearing.

## Methods

### Listeners with SSD and control listeners

Nineteen listeners with complete SSD (16–67 years; mean ± *SD*: 40.7 ± 16.7 years) and 15 control listeners (22–61 years; mean ± *SD*: 30.9 ± 12.4 years) participated in the present study. Table 1 lists the characteristics of listeners with SSD and indicates which listeners experienced listening with a bone-conduction device. To assess hearing loss in the better ear, we performed pure-tone audiometry at 0.125, 0.25, 0.5, 1, 2, 4, and 8 kHz. Hearing thresholds were thus obtained using standard procedures and standard equipment (Interacoustics AC 40 clinical audiometer, Interacoustics A/S, Assens, Denmark).

**Table 1:** **Audiometric characteristics of the listeners with SSD**.

**SSD patients**	**Age (y)**	**Side HL**	**Congenital acquired**	**Gender**	**Threshold dB HL 8 kHz**
P1	32	L	Congenital	M	0
P2	22	L	Congenital	M	10
P3	22	L	Congenital	M	5
P4	24	R	Congenital	V	10
P5	51	L	Congenital	M	65
P6^*^	46	L	Congenital	V	10
P7^*^	27	R	Congenital	M	5
P8	46	L	Congenital	V	5
P9^*^	16	L	Congenital	M	0
P10^*^	34	L	Congenital	M	35
P11	20	L	Congenital	V	0
P12	67	L	Acquired	M	70
P13	38	R	Acquired	V	20
P14^*^	53	R	Acquired	V	40
P15^*^	63	L	Acquired	V	5
P16	34	L	Acquired	M	30
P17	51	L	Acquired	M	40
P18	67	R	Acquired	M	55
P19	60	L	Acquired	M	60

* Indicates listeners who experienced listening with a bone-conduction device.

### Mold in the better ear

The SSD listeners were tested in two hearing conditions that were presented in randomized order: (i) monaural hearing; (ii) monaural hearing with a custom-made mold, fabricated from rubber casting material (Otoform Otoplastik—K/c; Dreve, Unna, Germany), inserted in the pinna of the better-hearing ear without obstructing the ear canal.

All control listeners were tested under normal hearing conditions, and after altering their pinna-cues with custom-made molds in both pinna.

### Stimuli

Listeners were asked to localize (i) low-pass (LP; 0.5–1.5 kHz); (ii) high-pass (HP; 3–20 kHz), and (iii) broadband (BB; 0.5–20 kHz) filtered Gaussian white noises. Spectral cues are minimal for LP noises (Middlebrooks and Green, 1991; Middlebrooks, 1992; Frens and Van Opstal, 1995; Blauert, 1997; Van Wanrooij and Van Opstal, 2004, 2007), and we therefore hypothesized that LP noises could not be localized in azimuth at all by SSD listeners.

BB and HP stimuli were chosen to maximize the use of potential spectral-shape cues provided by the pinna of the better-hearing ear. BB and HP stimuli had randomly-selected sound levels in the range 45–65 dB SPL. LP noises were interleaved with the BB and HP stimuli, and only presented at a level of 55 dB SPL. To minimize measurement time and because the attenuation of sound level by the head is not very effective for LP noises, we decided not to rove the levels of the LP stimuli.

All stimuli had 150-ms duration, 5-ms sine- and cosine-squared on- and offset ramps and a flat spectrum level within their pass bands. Sounds were digitally generated in Matlab (The MathWorks) at a sampling rate of 50 kHz, and were delivered through a BB loudspeaker, moved by a computer-controlled motorized system at a distance of 1.15 m from the listener's head. Stimulus coordinates for BB and HP stimuli ranged from −85° to +85° in azimuth and from −30° to +30° in elevation. LP stimuli were presented at 0° in elevation.

### Setup

For a detailed description of the setup see Bremen et al. (2010). Briefly, we ensured that listeners could only use acoustic information to localize sounds by testing directional hearing in a completely dark, sound-attenuated room. Horizontal and vertical head-movement components were recorded with the magnetic search-coil induction technique (Robinson, 1963; Hofman and Van Opstal, 1998). Listeners pointed with a head-fixed laser pointer, which projected onto a small (1 cm^2^) black plastic plate positioned in front (40 cm) of the listener's eyes. Listeners were asked to point the laser dot as fast and as accurately as possible in the perceived sound direction after stimulus exposure. Listeners were observed continuously by the experimenter with an infrared camera, but did not receive any feedback about their performance during the experiments.

### Paradigm

The experimental session started with a brief visual calibration experiment to establish the off-line mapping of the coil signals onto known target locations. After this, listeners performed a brief practice session containing 10 trials to become familiar with the head-movement response procedure.

During the sound-localization experiments the listener first fixated on an LED that was located at 0° azimuth and 0° elevation and then triggered the start of the trial by pressing a button. Between 150 and 300 ms the LED disappeared, and 200 ms later the sound stimulus was presented. After stimulus exposure the listener had to direct the head-fixed laser pointer as fast and accurately as possible, by making a rapid head movement toward the apparent sound direction.

### Data analysis

We analyzed the azimuth (α) responses separately for each stimulus condition (LP, HP, and BB noises) and for each listener. We determined the best linear fit (based on the mean-squared error criterion) of the stimulus-response relationship (pooled across presentation levels and elevation angles for HP and BB noises):

(1) $$\alpha_{RESP}=b+g\;\cdot \;\alpha_{STIM}$$

where α_*RESP*_ is the response azimuth (in degrees), α_*STIM*_ is the stimulus azimuth (in degrees), *b* is the response bias (in degrees), and *g* the response gain (dimensionless). We also computed Pearson's correlation coefficient between fit and data, as well as the coefficient of determination (*r*^2^). To dissociate the potential contribution of the proximal sound level, *L*, from that of the actual stimulus location, we performed a partial correlation analysis:

(2) $${\hat{\alpha }}_{RESP}=p\;\cdot \;{\hat{\alpha }}_{STIM}+q\;\cdot \hat{L}$$

with *p* as the dimensionless azimuth coefficient and *q* as the dimensionless proximal sound-level coefficient; each determines to what extent sound-source azimuth or proximal sound level explains the observed responses. Variables α_*RESP*_, α_*STIM*_ and *L* were transformed into their (dimensionless) z-scores $\hat{x}$:

(3) $$\hat{x}\equiv \frac{x-\mu_{x}}{\sigma_{x}}$$

with *x* the variable to be z-transformed, μ_*x*_ its mean, and σ_*x*_ its standard deviation (resulting in $\hat{\alpha }$_*RESP*_, $\hat{\alpha }$_*STIM*_, and $\hat{L}$). We determined proximal sound level *L* by correcting the free-field presentation levels of the stimuli with the frequency- and azimuth-dependent attenuation produced by the HSE.

The HSE was derived for BB noises from the best fit of free-field HSE measurements of four listeners (Van Wanrooij and Van Opstal, 2004). For HP and BB noises the HSE can vary between −15 and +15 dB over the entire azimuth range, for LP noises the HSE is less pronounced.

For the elevation (ε) responses to BB and HP noises the best linear fits of the stimulus-response relationships were also determined.

(4) $$\varepsilon_{RESP}=b+g\;\cdot \;\varepsilon_{STIM}$$

ε_*RESP*_ and ε_*STIM*_ are the response elevation and stimulus elevation in degrees, *b* is the response bias (in degrees) and *g* the response gain (dimensionless).

## Results

### High-frequency hearing loss

Normal hearing thresholds (defined as 20 dB HL or better) in the functioning ear were confirmed in all listeners with SSD (*n* = 19) for frequencies up to 4 kHz. At 8 kHz 11 SSD listeners demonstrated normal hearing. The other SSD listeners demonstrated thresholds ≥20 dB HL, with six listeners demonstrating thresholds ≥40 dB HL (see Table 1).

In the group of control listeners (*n* = 15), two older listeners (age 56 and 61 years) suffered from a symmetric hearing loss at 8 kHz (thresholds ≥ 40 dB HL). All other control listeners demonstrated normal hearing thresholds between 500 Hz and 4 kHz, and thresholds of 40 dB HL, or better, for 8 kHz.

### Effect of stimulus bandwidth

**Figure 1:**
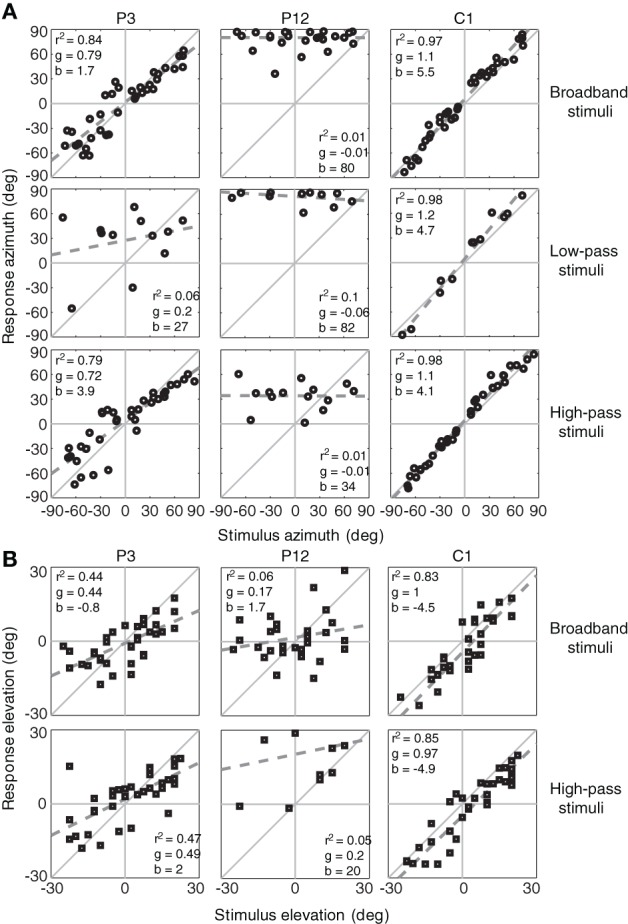
**Sound-localization responses for SSD listener with a thresholds at 8 kHz < 40 dB HL (P3), a SSD listener with a thresholds at 8 kHz ≥ 40 dB HL (P12) and a control listener (C1).** Responses are plotted for the BB, HP, and LP stimuli in azimuth **(A)** and elevation **(B)**. The dashed gray line denotes the linear regression fit. Note the high degree of variation in monaural localization abilities of the listeners with SSD. Listener P3 had fairly good localization of BB and HP stimuli. *r*^2^, coefficient of determination, *g*, response gain, *b*, bias.

Figure 1A shows the stimulus-response relations in azimuth for a control listener (C1), and two listeners with SSD at their left side (P3 and P12), for BB, LP, and HP stimuli. For the BB and HP stimuli responses for the presentation levels (45, 55, and 65 dB SPL) were pooled. The dashed lines represent the best-fit linear regression lines (Equation 1) on the azimuth response components. The control listener (right-hand column) could accurately localize stimuli for all conditions as is indicated by *r*^2^ values and gains close to 1. Note that perfect localization would mean that all individual responses would exactly be on the diagonal with slope +1.0 (with parameters: *r*^2^ = 1, *g* = 1, *b* = 0). Listener P3 with SSD demonstrated good localization performance for BB and HP stimuli (*r*^2^ > 0.79; *g* > 0.72; *b* between 0° and 4°). In contrast, listener P12 with SSD demonstrated poor sound-localization abilities. This listener perceived the stimuli mainly at the hearing side, which resulted in a considerable leftward bias (*b* = 80° for BB stimuli), and small coefficients of determination (*r*^2^ < 0.10) for all stimuli and conditions. The hearing thresholds at 8 kHz in the better ear are listed in Table 1. Listener P3 demonstrated a 8 kHz hearing threshold of 5 dB HL, P12 demonstrated a 8 kHz threshold of 70 dB HL. Because of the high-frequency hearing loss listener P12 did not detect all stimuli.

Figure 1B shows the stimulus-response relations in elevation (Equation 4) for the same listeners. Listener P3, with better horizontal sound localization abilities than listener P12, demonstrated also better elevation performance (*g* > 0.44 vs. *g* < 0.2).

**Figure 2:**
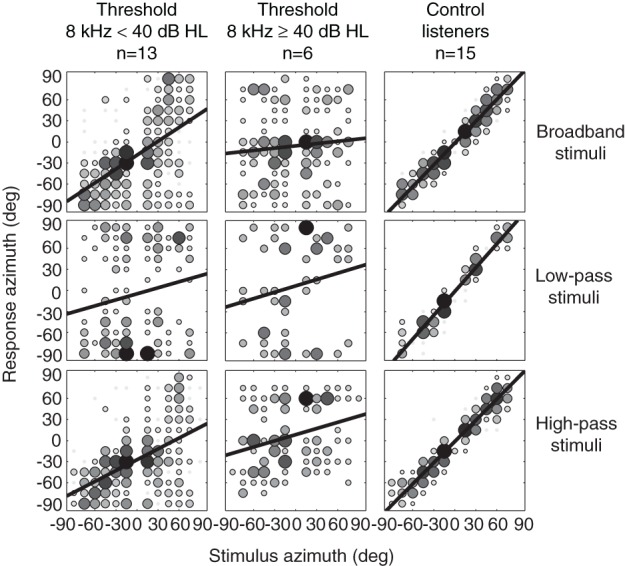
**Azimuth stimulus-response relationships for BB, LP, and HP noise burst pooled for SSD listeners with 8 kHz thresholds below 40 dB HL (left hand column), SSD listeners with 8 kHz thresholds higher than 40 dB HL (middle column), and control listeners (right hand column).** Black bold lines denote best-fit regression lines over the pooled data. Grayscale and size of the data points indicates the number of responses on that location. Black indicates a larger number of responses than white.

Figure 2 shows the pooled azimuth stimulus-response relations of all control listeners (*n* = 15), all SSD listeners with 8 kHz thresholds below 40 dB HL (*n* = 13), and SSD listeners with 8 kHz thresholds higher than 40 dB HL (*n* = 6), for BB, LP and HP stimuli. If the right ear was the deaf ear, data are presented without modification. If the left ear was the deaf ear, data of left and right ears were swapped before pooling the data. The figure demonstrates that listeners without high-frequency hearing loss outperformed listeners with 8 kHz thresholds higher than 40 dB HL, for BB and HP sounds. The figure hints at the possibility that SSD listeners with 8 kHz thresholds below 40 dB HL were able to use spectral pinna-cues, as they could localize the BB and HP stimuli in azimuth, but were not able to localize the LP sounds. Listeners with 8 kHz thresholds higher than 40 dB HL were equally poor in localization of BB, LP, and HP stimuli.

**Figure 3:**
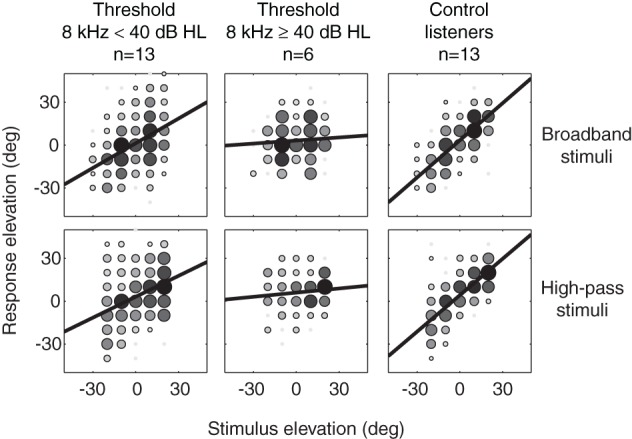
**Elevation stimulus-response relationships for BB and HP noise burst pooled for SSD listeners with 8 kHz thresholds below 40 dB HL (left hand column), SSD listeners with 8 kHz thresholds higher than 40 dB HL (middle column) and control listeners (*n* = 13, right hand column).** Black bold lines denote best-fit regression lines over the pooled data. Grayscale and size of the data points indicates the number of responses on that location. Black indicates a larger number of responses than white.

Figure 3 shows the pooled stimulus-response relations in elevation. Listeners with 8 kHz thresholds below 40 dB HL outperformed listeners with 8 kHz thresholds higher than 40 dB HL. The figure shows that SSD listeners with 8 kHz thresholds below 40 dB HL were able to use spectral pinna-cues for the localization of BB and HP stimuli in elevation. Listeners with 8 kHz thresholds higher than 40 dB HL were equally poor in localization of BB and HP stimuli. Two control listeners with high-frequency hearing loss (threshold 8 kHz > 40 dB HL) were not included in the pooled elevation stimulus-response relations (right hand column).

### Contribution of spectral cues

**Figure 4:**
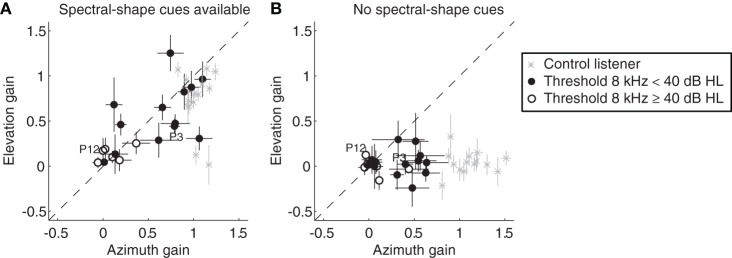
**Response elevation gain for BB stimuli plotted against the azimuth gain.** Data from all control listeners (gray crosses), listeners with SSD with 8 kHz thresholds below 40 dB HL (filled circles) and SSD listeners with 8 kHz thresholds higher than 40 dB HL (open circles) are presented when spectral-shape cues were available **(A)**, and when spectral-shape cues were reduced by molds **(B)**. Error bars denote ± 1 SE of the azimuth and elevation regression coefficients. Data points from the two SSD listeners depicted in Figure 1 (P3 and P12), are indicated in the figure. Data are pooled across presentation levels. Note the two clear outliers in the control group. These two listeners demonstrated bilateral high-frequency hearing loss (8 kHz thresholds higher than 40 dB HL).

Figure 4 plots response azimuth localization gains for BB stimuli against response elevation gains for 13 SSD listeners with 8 kHz thresholds in the hearing ear below 40 dB HL (filled symbols), six SSD listeners with 8 kHz thresholds above 40 dB HL (open circles), and the 15 control listeners (crosses).

Figure 4A shows the gains for the listeners with SSD in the monaural condition, and for the normal hearing control listeners (spectral-shape cues are available). Listeners with SSD demonstrated considerable variability in performance, and there was a clear correlation between azimuth gains and elevation gains (*r* = 0.83, *p* < 0.01). The SSD listeners with 8 kHz thresholds below 40 dB HL demonstrated higher azimuth and elevation gains than the SSD listeners with thresholds above 40 dB HL. The latter group of listeners had both gains close to zero, indicating poor directional hearing performance in both azimuth and elevation. The *r*^2^ were also small (<0.4, data not shown). The far majority of control listeners had azimuth and elevation gains that were close to the ideal value of one. The two older control listeners with high-frequency hearing loss demonstrated small elevation gains, confirming earlier reports of deteriorated vertical sound localization performance in the elderly (Otte et al., 2013).

Figure 4B shows the resulting azimuth and elevation gains when the molds reduced the spectral-shape cues (*r* = 0.2, *p* = 0.87). Note that the SSD listeners with a relatively low high-frequency hearing loss demonstrated a clear deterioration in their sound localization performance in both directions. Molds in the pinnae of control listeners only affected their elevation performance. This deterioration of sound localization abilities in elevation, after altering the pinna-cues with custom-made molds in both pinna, has been reported previously (Oldfield and Parker, 1984).

### Contribution of high-frequency hearing loss

**Figure 5:**
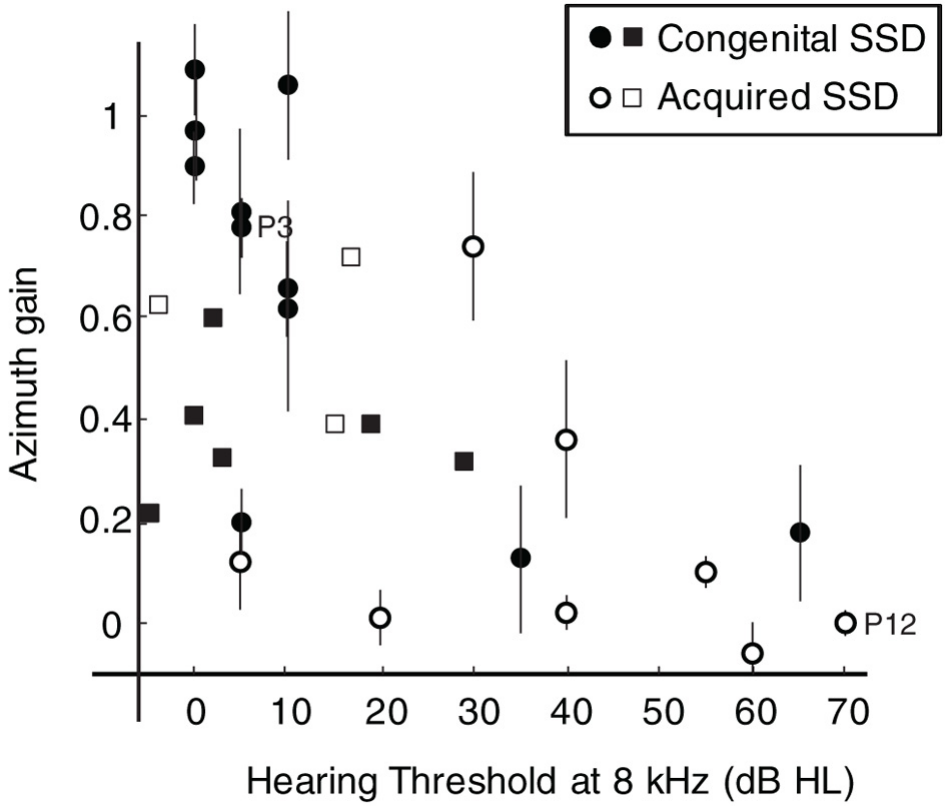
**Response azimuth gain for BB stimuli plotted against the hearing threshold at 8 kHz for listeners with congenital SSD (filled circles) and listeners with acquired SSD (open circles).** Error bars denote ± 1 SE of the azimuth regression coefficient. Data points from SSD listeners P3 and P12 are emphasized in the figure. For comparison data (squares) of nine listeners with SSD from a study performed by Van Wanrooij and Van Opstal (2004) are plotted in the figure.

Figure 5 illustrates the effect of high-frequency (8 kHz) hearing loss on the localization performance, of BB noises, of SSD listeners in the horizontal plane. When the hearing loss at 8 kHz exceeds about 30 dB HL the azimuth gains are always small (*g* < 0.4). Good high-frequency hearing in the only hearing ear appears to be an important requirement for adequate sound localization performance. The variation in localization performance is not explained by the onset of unilateral deafness (congenital vs. acquired). In addition we also included the data of 9 listeners with SSD from the study of Van Wanrooij and Van Opstal (2004; squares). This figure clearly shows that almost half of the subjects with 8 kHz thresholds below 40 dB HL demonstrate poor sound localization abilities.

Elevation gains also clearly deteriorate with increasing high-frequency hearing loss. For all subjects with 8 kHz thresholds above 40 dB HL elevation gains were small.

### Effect of sound level on localization performance

**Figure 6:**
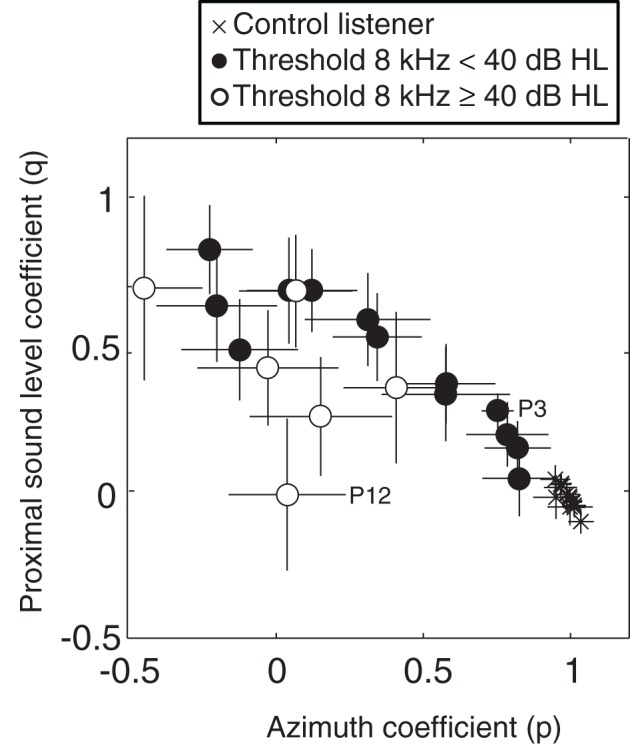
**Multiple linear regression analysis of azimuth localization performance for BB stimuli of SSD listeners with 8 kHz thresholds below 40 dB HL (filled circles), SSD listeners with 8 kHz thresholds higher than 40 dB HL (open circles) and control listeners (crosses).** The coefficients for proximal sound level (*q* in Equation 2) and azimuth (*p* in Equation 2) are plotted against one another for each listener. Error bars denote ± 1 *SD* of the azimuth and intensity regression coefficients, respectively. Data points from SSD listeners P3 and P12 are emphasized in the figure. For clarity, some data points are slightly shifted.

Figure 6 shows the partial correlation coefficients for azimuth (*p* in Equation 2) and for the proximal sound level (*q* in Equation 2) for the BB stimuli, for SSD listeners (circles) and control listeners (crosses). These partial correlation coefficients reveal the relative contributions of the actual target azimuth and the perceived sound level at the hearing ear to their azimuth localization responses. For SSD listeners with an 8 kHz threshold below 40 dB, the contribution of proximal sound level varied systematically with the azimuth coefficient. Responses were more influenced by sound level when the (spectrally derived) estimate of azimuth was poor. Indeed, those SSD listeners typically perceived louder sounds on their hearing side. A similar effect of sound level on localization performance in cochlear-implant listeners has been reported by Majdak et al. (2011).

Listener P12 is the listener with the most severe high-frequency hearing loss (see Table 1). This listener did not detect all BB stimuli and therefore proximal sound level did not contribute to the localization performance.

Control listeners had their azimuth coefficients close to the ideal value of one, and the proximal sound level coefficient close to zero. When listeners can localize sounds on the basis of binaural difference cues they rely less on the HSE cue.

## Discussion

### Individual differences in sound localization performance

The present study demonstrates that SSD listeners without severe high-frequency hearing loss in their hearing ear can localize BB noises in the horizontal plane. Our data indicate that the amount of high-frequency hearing loss greatly influences the directional hearing abilities of SSD listeners (Figure 5). Colburn (1982) postulated that the etiology of subjects with unilateral total deafness (e.g., congenital vs. acquired), may be irrelevant for their localization abilities. In support of this idea, our data indicate that the variability in localization performance of SSD listeners can to a large extent be attributed to high-frequency hearing loss, and not to the onset of unilateral deafness (congenital vs. acquired). However, good high-frequency hearing (8 kHz thresholds <40 dB HL) does not always ensure good sound localization abilities. Even in the group of SSD listeners with 8 kHz thresholds below 40 dB HL almost half of the subjects demonstrate poor sound localization. This variation in sound localization performance can be related to several factors. Recently, Andéol et al. (2013) and Majdak et al. (2014) demonstrated that in listeners with normal hearing, non-acoustic factors like the perceptual ability to discriminate spectral shapes had a larger impact on the sound localization performance in elevation than cues provided by the listener-specific pinna-induced spectral-shape cues. These non-acoustic factors might also play a role in the azimuthal localization abilities of SSD listeners.

### Pinna-induced spectral-shape cues

Some listeners with SSD were able to use the spectral-pinna cues of their hearing ear for localization in azimuth. When the possibility to use spectral cues was disrupted by filling the pinna of their hearing ear with a mold, azimuthal localization deteriorated (Figure 4B). The spectral cues are specific for an individual listener and appear above about 4 kHz (Batteau, 1967; Middlebrooks and Green, 1991). BB noises can be localized in the vertical plane, because the brain can dissociate the elevation dependent pinna-induced spectral shape cues. Apparently, azimuth dependent changes in the spectral cues are used when the auditory system is deprived from binaural cues. Recently Otte et al. (2013) demonstrated that the pinna-induced spectral shape cues are changing during life because the ears keep growing, and that listeners adapt to this changing cues. A limitation of the present study is that we did not measure the spectral cues in terms of head-related transfer functions (HRTFs) or non-acoustic factors like the perceptual ability to discriminate spectral shapes (Andéol et al., 2013; Majdak et al., 2014) of the SSD listeners.

### Increasing number of treatment options for SSD

Studies have shown that children with SSD demonstrate worse language scores compared to their normal-hearing peers, and that they are at risk for learning problems in school (Lieu, 2013). There is increasing evidence that adults with SSD experience problems in social settings because of their disability in binaural processing (Wie et al., 2010).

The criteria for treatment of SSD are expanding, and more treatment options become available. One treatment option is to provide a contralateral routing of sound (CROS) device. These devices transmit sounds presented at the deaf side to the hearing ear. Currently, the two most commonly applied CROS interventions are the wireless conventional CROS hearing aid, and the percutaneous bone-conduction hearing device (Bosman et al., 2003). Although listeners with SSD have only a single functioning cochlea, and therefore bone-conduction would not restore binaural hearing, the bone-conduction device is offered more often as an option for rehabilitation (Spitzer et al., 2002; Hol et al., 2004; Newman et al., 2008; Grantham et al., 2012; Nicolas et al., 2012; Battista et al., 2013).

In several countries cochlear implantation has become a treatment option (Arndt et al., 2011; Kamal et al., 2012; Arnoldner and Lin, 2013), and it is even proposed to implant children with congenital SSD already at a young age (Tzifa and Hanvey, 2013). Potentially, this option can lead to binaural hearing.

## Conclusion

The present study emphasizes the importance of a precise evaluation of the monaural hearing abilities of listeners with SSD, especially at the higher frequencies for which the spectral-shape cues become unambiguous for sound localization. Some SSD listeners were using monaural pinna-induced spectral-shape cues of their hearing ear, for localization of BB noises in both azimuth and elevation. Because spectral cues are minimal for LP noises (Middlebrooks, 1992; Blauert, 1997) these stimuli could not be localized by SSD listeners. For clinicians it might be important to understand the factors affecting the localization performance of SSD listeners in order to give the hearing impaired the best advice in case of desired treatment.

### Conflict of interest statement

The authors declare that the research was conducted in the absence of any commercial or financial relationships that could be construed as a potential conflict of interest.

## Abbreviations

BB: broadband
HP: high-pass
HRTFs: head-related transfer functions
HSE: head-shadow effect
ILDs: interaural level differences
ITDs: interaural time differences
LP: low-pass
MAE: mean absolute error
SSD: single-sided deaf(ness).
